# Adult-onset combined oxidative phosphorylation deficiency type 14 manifests as epileptic status: a new phenotype and literature review

**DOI:** 10.1186/s12883-023-03480-4

**Published:** 2024-01-02

**Authors:** Xu Zhang, Feng Xiang, Desheng Li, Fei Yang, Shengyuan Yu, Xiangqing Wang

**Affiliations:** Department of Neurology, The First Medical Center of Chinese PLA General Hospital, 28# Fuxing Road, Beijing, People’s Republic of China

**Keywords:** Combined oxidative phosphorylation deficiency type 14, FARS2 gene, Adult, Epileptic status

## Abstract

**Background:**

Combined oxidative phosphorylation deficiency (COXPD) is a severe disorder with early onset and autosomal recessive inheritance, and has been divided into 51 types (COXPD1–COXPD51). COXPD14 is caused by a mutation in the FARS2 gene, which encodes mitochondrial phenylalanyl-tRNA synthetase (mt-PheRS), an enzyme that transfers phenylalanine to its cognate tRNA in mitochondria. Since the first case was reported in 2012, an increasing number of FARS2 variations have been subsequently identified, which present three main phenotypic manifestations: early onset epileptic encephalopathy, hereditary spastic paraplegia, and juvenile-onset epilepsy. To our knowledge, no adult cases have been reported in the literature.

**Methods:**

We report in detail a case of genetically confirmed COXPD14 and review the relevant literature.

**Results:**

Approximately 58 subjects with disease-causing variants of FARS2 have been reported, including 31 cases of early onset epileptic encephalopathy, 16 cases of hereditary spastic paraplegia, 3 cases of juvenile-onset epilepsy, and 8 cases of unknown phenotype. We report a case of autosomal recessive COXPD14 in an adult with status epilepticus as the only manifestation with a good prognosis, which is different from that in neonatal or infant patients reported in the literature. c.467C > T (p.T156M) has been previously reported, while c.119_120del (p.E40Vfs*87) is novel, and, both mutations are pathogenic.

**Conclusions:**

This case of autosomal recessive COXPD14 in an adult only presented as status epilepticus, which is different from the patients reported previously. Our study expands the mutation spectrum of FARS2, and we tended to define the phenotypes based on the clinical manifestation rather than the age of onset.

## Introduction

Enzyme deficiencies in the oxidative phosphorylation (OXPHOS) system can be caused by mutations in mitochondrial or nuclear DNA. Ninety-nine percent of mitochondrial proteins are encoded by nuclear genes, and mutations in the gene encoding aminoacyl tRNA synthetase can lead to combined oxidative phosphorylation deficiency (COXPD). Mitochondrial aminoacyl-tRNA synthetase proteins (mt-aaRSs) are a group of nuclear-encoded enzymes that facilitate conjugation of each of the 20 amino acids to its cognate tRNA molecule [1]. To date, COXPD has been divided into 51 types (COXPD1–COXPD51), based on different disease-causing genes. COXPD14, caused by mutation of the FARS2 gene, mainly manifests as three phenotypes: early onset epileptic encephalopathy [2–11], hereditary spastic paraplegia [10, 12–18], and juvenile onset epilepsy [19–21]. FARS2 is a nuclear gene located on the short arm of chromosome 6 (6p25.1) that consists of seven exons spanning over 510 kb [7]. Since the first case was reported in 2012 [2], a total of 55 subjects have been reported in the English-language literature, including seven Chinese cases [11, 13, 20]. In addition, three cases have been reported in the Chinese literature [22, 23].

Here, we present the clinical, and radiological findings and molecular genetics of a Chinese adult affected by COXPD14 and found two compound heterozygous variants in FARS2 by whole-exome sequencing (WES), which further expanded the molecular and phenotypic spectrum of COXPD14 caused by genetic defects in FARS2. To our knowledge, the present study is the first to report adult onset COXPD14. In addition, we retrospectively reviewed and summarized the clinical and radiological findings and molecular data of patients with FARS2 deficiency.

## Clinical report

### Clinical manifestations

The index patient, a female, was born to nonconsanguineous Chinese parents with normal neurodevelopment and no risk factors for epilepsy. Two healthy children were delivered by two cesarean sections within 5 years before the onset of the disease. When she was 27 years old and 3 months after the second delivery, she was observed to have a first seizure characterized by status epilepticus, with a total of five seizures of status epilepticus. Each attack induced fatigue, mood fluctuations, and lack of sleep. All seizures showed tonic clonus and loss of consciousness, followed by right upper limb jitter, and each attack was treated with endotracheal intubation, a ventilator, and sedative drugs. No headache, vision loss, diplopia, limb weakness, or myoclonus was observed. After the onset of the disease, she began to experience personality changes, irritability, unresponsiveness, memory decline, and computational decline. Upon admission, physical examination showed a sluggish reaction, slightly poor listening and understanding, verbal expression disorders, decreased near and far memory, and decreased numeracy. The location, time, and character orientation decreased. The cranial nerve examination was normal. There was no apparent muscle atrophy. Both muscle tone and strength were normal. No abnormalities were found in the sensory system examination, and bilateral plantar responses were flexor. The patient was prescribed levetiracetam, sodium valproate, oxcarbazepine, and phenobarbital to control seizures.

**Fig. 1:**
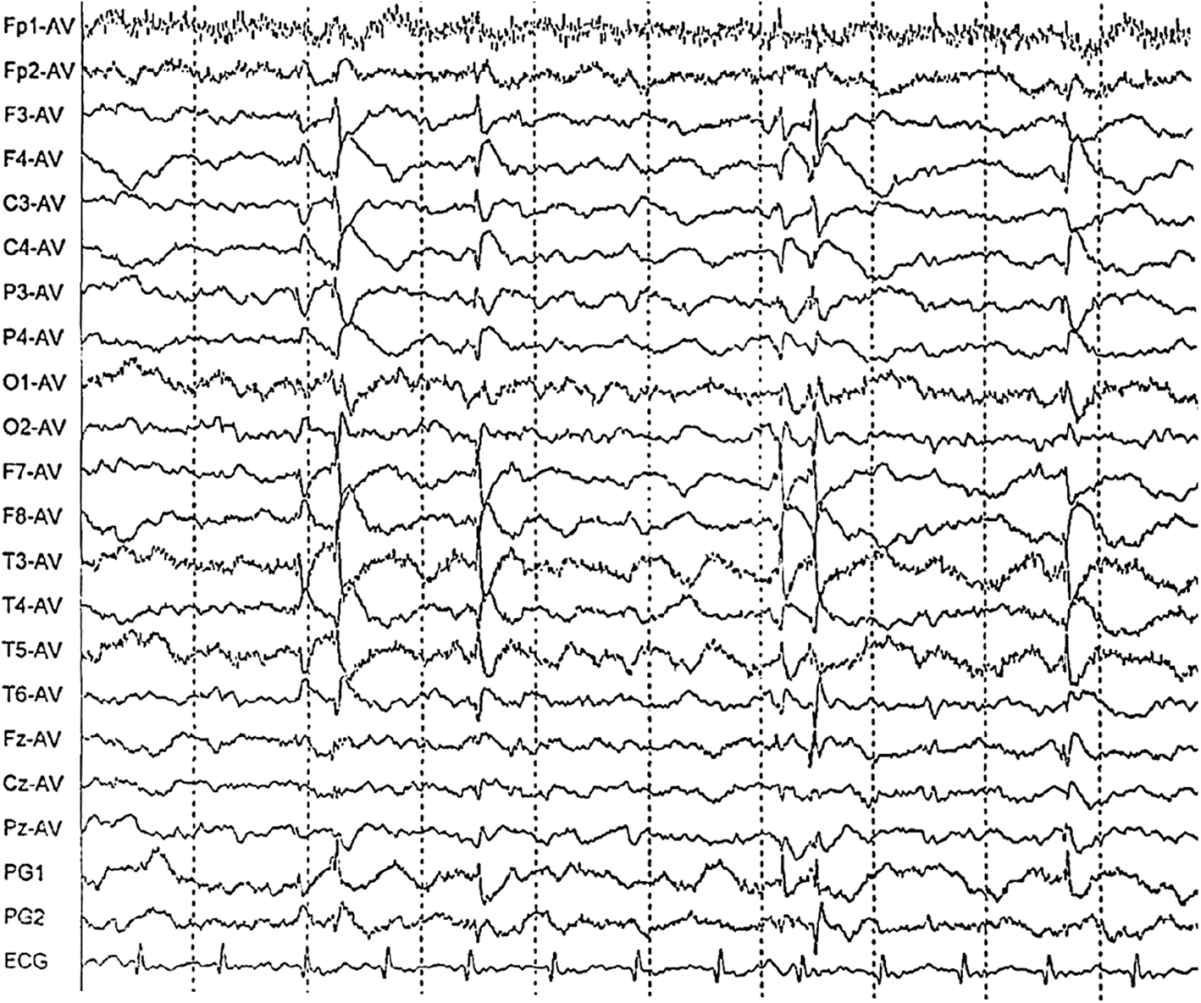
EEG showed unilateral, persistent spike-slow complex waves in the left frontal area, subfrontal area and middle temporal area

We performed an electroencephalogram (EEG) that immediately showed unilateral, persistent, spike-slow complex waves in the left frontal, subfrontal, and middle temporal areas (Fig. 1). Laboratory examination indicated that antibodies related to autoimmune encephalitis and paraneoplastic antibodies in the serum or cerebrospinal fluid were negative. Thyroid function, rheumatic immune indices, and tumor marker levels were negative. The lactate/pyruvate ratio in the serum and lactate levels in the cerebrospinal fluid were normal. The routine indices of the cerebrospinal fluid and the biochemical, immune, and smear tests were normal.

**Fig. 2:**
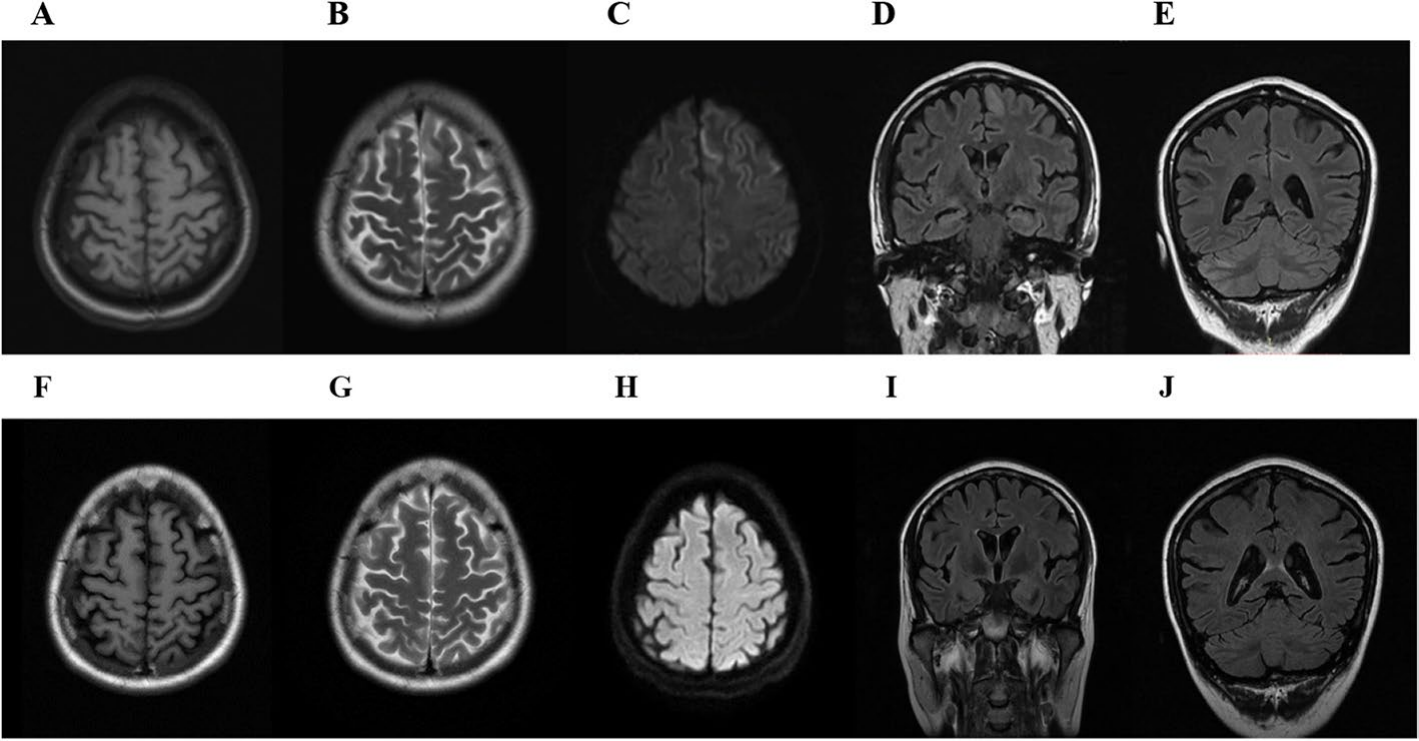
Brain MRI of the patient on 10–2020. **A**-**D** Abnormal signal in the left frontal lobe (white arrow). **E** Hyperintensity of the right cerebellum on FLAIR (yellow arrow). Brain MRI on 11–2020. **F**-**J** All the above abnormal signals disappeared

**Fig. 3:**
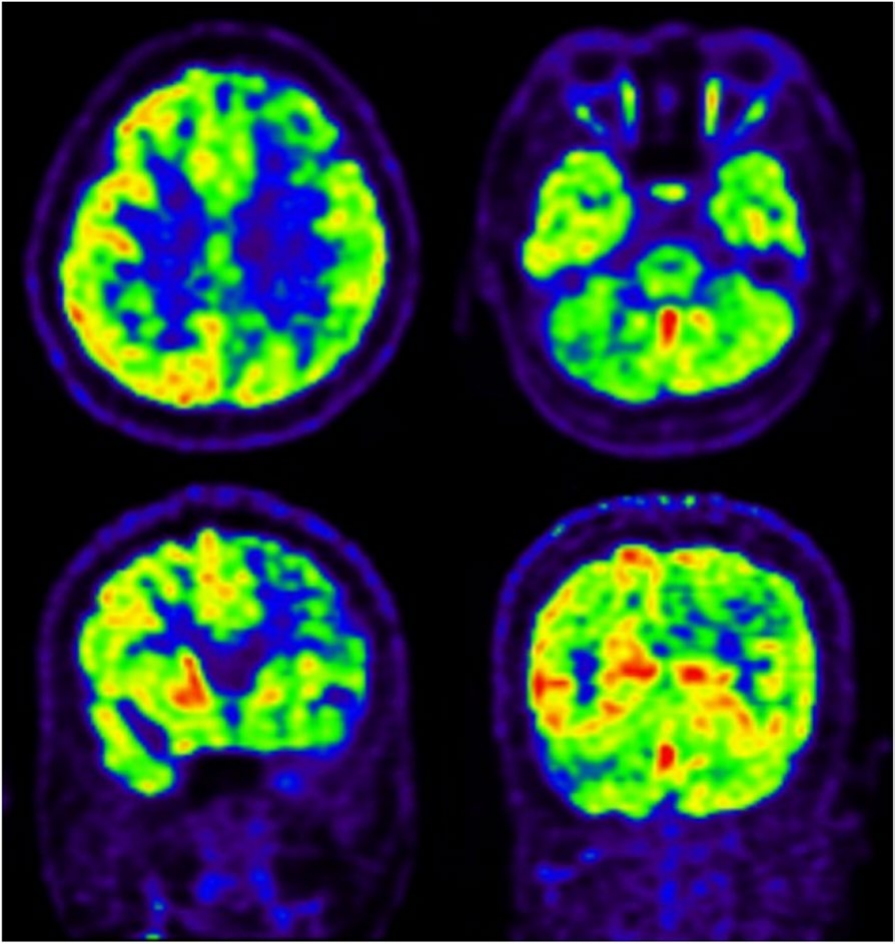
PET-CT of the patient on 10–2020, showed diffuse decreased metabolic heterogeneity in the bilateral cerebral hemispheres, mainly in the left frontal lobe, and a diffuse decrease in metabolism in the right cerebellum, which was considered CCD

**Fig. 4:**
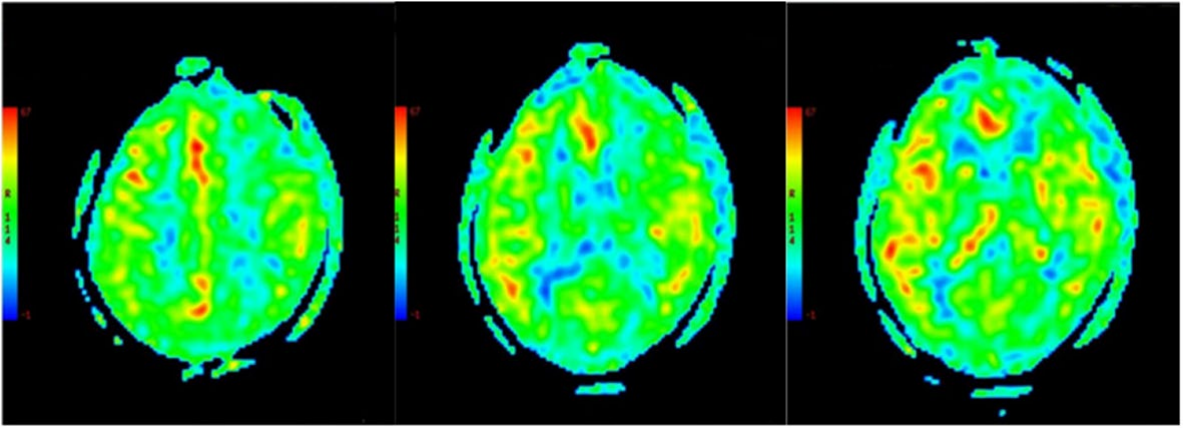
CBF, slight decrease in the left frontal lobe considered to be a secondary change

After admission, craniocerebral magnetic resonance imaging (MRI) revealed that the bilateral frontal lobe volume had slightly decreased, and the thin corpus callosum and lateral ventricle were slightly enlarged. For comparison, craniocerebral MRI one month before admission showed abnormal signals in the left frontal lobe and right cerebellum, and hyperintensity on diffusion-weighted imaging (DWI) and fluid-attenuated inversion recovery (FLAIR) signal abnormalities, which disappeared after 1 month (Fig. 2). At the same time, positron emission tomography-CT (PET-CT) at 10–2020 revealed diffuse decreased metabolic heterogeneity in the bilateral cerebral hemispheres, mainly in the left frontal lobe, and a diffuse decrease in metabolism in the right cerebellum, considering the change in crossed cerebellar diaschisis (CCD) (Fig. 3). After admission, cerebral perfusion weight imaging indicated a slight decrease (CBF) in the left frontal lobe that was considered a secondary change (Fig. 4).

### Molecular genetic analysis

**Fig. 5:**
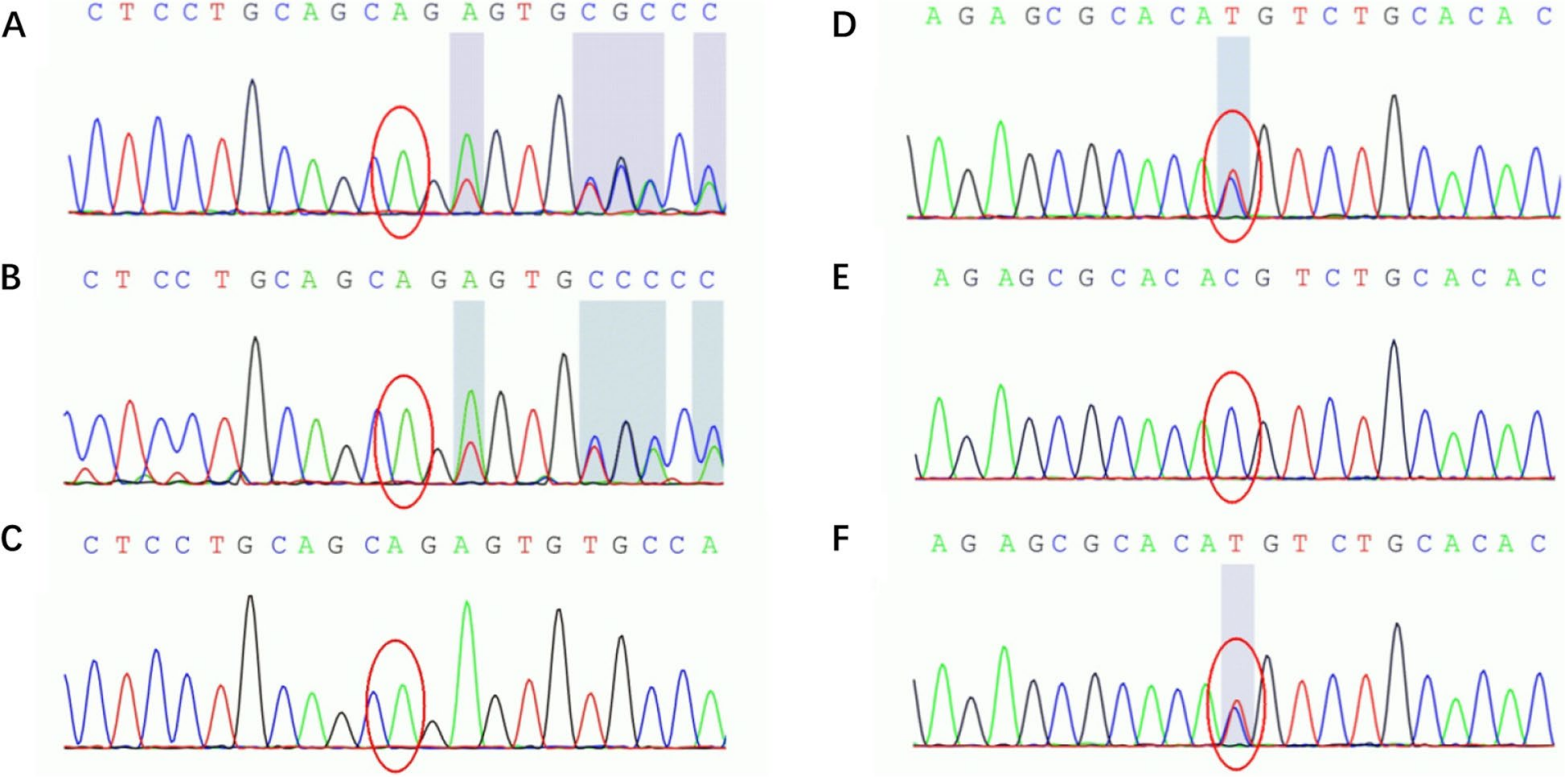
Partial DNA sequence chromatograms of FARS2. The red circles represent the location of the variants c.119_120 del (**A**) and c.467C > T (**D**) in the FARS2 gene, inherited from the father (**B**) and mother (**F**)

Genetic testing of the mitochondria revealed no abnormalities. The FARS2 gene (NM_006567.3 exon2) complex heterozygous mutation was identified using whole exome sequencing (WES) (Fig. 5). The c.119_120 del (deletion mutation), resulting in amino acid change p. E40Vfs * 87 (terminated after—87 b frameshift mutation), has not been reported in the HGMD pro database [24]. c.467C > T (cytosine > thymine), resulting in the amino acid change p.T156M (threonine > methionine), was reported as a pathogenic variation associated with epileptic encephalopathy with unknown onset [25, 26]. Family validation showed that the heterozygous mutation was a compound heterozygous mutation from the parents, which was consistent with the rule of autosomal recessive inheritance. According to the American College of Medical Genetics and Genomics (ACMG) guidelines [27], the mutation c.119_120del can be rated as a likely pathogenic mutation (PVS1 + PM2; PVS1: frameshift mutation of genes with LOF mechanism; PM2: no report in normal human variation database), and variation c.467C > T can be rated as a likely pathogenic variation (PM2 + PM3_Strong; PM2: recessive gene variation has a low population frequency; PM3_Strong: trans distribution with two suspected pathogenic variants) (Table 1). In addition, Mutation-Taster and FATHMM software proved that the above two sites were pathogenic mutations. In summary, the complex heterozygous variation observed in this patient is theoretically pathogenic. The final diagnosis was confirmed to be combined oxidative phosphorylation defect type 14 (COXPD14).

**Table 1:** Pathogenicity analysis of the two FARS2 variants

Gene	Mutations	Amino acid change	Status	Normal population carrying rate	Validation of pedigree	ACMG Rating of variation	HGMD Transcriptional version of the gene subregion	References
FARS2	c.119_120del	p.E40Vfs*87	Novel	-	Father source	Likely pathogenic	NM_006567.3 exon2	-
FARS2	c.467C > T	p.T156M	Known	0.0000886	Mother source	Likely pathogenic	NM_006567.3 exon2	PubMed_ID:27652284

*ACMG* American College of Medical Genetics and Genomics, *HGMD* Human Gene Mutation Database

## Review of COXPD14

Here, we summarize all reported variations in FARS2. We used “FARS2,” “COXPD14” and “combined oxidative photosynthesis defect type 14” as keywords to search for relevant literature in biomedical literature database of the National Center for Biotechnology (NCBI) and China national knowledge infrastructure (CNKI). Analysis of the data of patients with FARS2 gene mutations reported before February 2023 was performed. The genetic and clinical features of previously reported cases of FARS2 variants are summarized in Table 2 according to the literature review. Approximately 58 subjects with disease-causing variants of FARS2 have been reported, including 31 cases of early-onset epileptic encephalopathy, 16 cases of hereditary spastic paraplegia, 3 cases of juvenile-onset epilepsy, and 8 cases of unknown phenotypes.

**Table 2:** Overview of reported FARS2 deficiency subjects [2–23]

Subject	Mutations	Gender	Onset age (outcome)	Ethnicity	Consanguinity	Developmental delay	Elevated lactate	Seizure	MRI findings	References
**Early-onset epileptic encephalopathy**										
1	c.431A > G (p.Y144C)	F	35 days (died at 20 months)	Arab	Yes	Yes	Yes	Uncontrolled myoclonic epilepsy	Cortical atrophy, widened sulci and fluid accumulation (at 1.5 years)	[2]
2	c.431A > G (p.Y144C)	M	NA (died before 3 months)	Arab	Yes	Yes	NA	Uncontrolled myoclonic epilepsy	NA	[2]
3	c.431A > G (p.Y144C)	M	NA (died before 3 months)	Arab	Yes	Yes	NA	Uncontrolled myoclonic epilepsy	NA	[2]
4	c.986 T > C (p.I329T), c.1172A > T (p.D391V)	F	2 days (died at 8 months)	Finnish	Yes	Yes	Yes	Myoclonic epilepsy, Multifocal seizures, Refractory	Normal at 4 days, extensive cortical atrophy with slight bilateral signal increase in the putamina at 3 months	[3]
5	c.986 T > C (p.I329T), c.1172A > T (p.D391V)	F	4 days (died at 21 months)	Finnish	Yes	Yes	NA	Refractory	NA	[3]
6	c.973G > T (p.D325Y), chr 6p25.1 del	M	6 months (alive at 30 months)	British	No	Yes	NA	Infantile spasms, focal seizures	Symmetrical subcortical white matter lesions. Thinning of corpus callosum	[4]
7	c.1156C > G (p.R386G) chr6: del	M	3 months (alive at 3 years)	Romanian	No	Yes	Yes	Infantile spasms (3 months), Seizure free (20 months)	severe brain atrophy, hyperintensity of T2 signal abnormalities in hemispheric white matter and dentate nuclei, thin corpus callosum	[5]
8	c.925G > A (p.G309S)	M	3 months (alive at 3 years)	Korean	No	Yes	Yes	GTC, SE, myoclonic	diffuse brain atrophy at 3 months; Progression of atrophic changes and myelination delay at 6 months	[6]
9	c.925G > A (p.G309S)	F	4 months (alive at 17 months)	Korean	No	Yes	No	Myoclonic at right hand, GTC, SE	Thin corpus callosum and generalized brain atrophy	[6]
10	c.925G > A (p.G309S)	M	4 months (died at 8 months)	Korean	No	Yes	Yes	Infantile spasms, SE	Mild brain atrophy	[6]
11	c.925G > A (p.G309S)	F	3 months (died at 44 months)	Korean	No	Yes	Yes	GTC, focal clonic	Mild brain atrophy	[6]
12	c.431A > G (p.Y144C)	F	4 months (died at 23 months)	Arab	Yes	Yes	Yes	Seizures of unknown type	Brain atrophy, thin corpus callosum	[7]
13	c.431A > G (p.Y144C)	F	40 days (died at 3 months)	Arab	Yes	Yes	Yes	Seizures of unknown type	Brain atrophy	[7]
14	c.431A > G (p.Y144C)	M	1 month (alive at 2 years)	Arab	Yes	Yes	Yes	Seizures of unknown type	Brain atrophy, thin corpus callosum	[7]
15	c.431A > G (p.Y144C)	F	2 months (NA)	Arab	NA	Yes	Yes	Seizures of unknown type	NA	[7]
16	c.431A > G (p.Y144C)	F	1 month (alive at 1 years)	Arab	No	Yes	Yes	Seizures of unknown type	Brain atrophy, thin corpus callosum	[7]
17	c.431A > G (p.Y144C)	M	1 month (died at 3 months)	Arab	Yes	Yes	Yes	Seizures of unknown type	thin corpus callosum	[7]
18	c.431A > G (p.Y144C)	F	1 month (alive at 13 months)	Arab	No	Yes	Yes	Seizures of unknown type	Brain atrophy, thin corpus callosum	[7]
19	c.431A > G (p.Y144C)	F	5 month (died at 2 years)	Arab	Yes	Yes	Yes	Seizures of unknown type	Brain atrophy, thin corpus callosum	[7]
20	c.431A > G (p.Y144C)	F	1 month (alive at 4.5 months)	Arab	Yes	Yes	Yes	Seizures of unknown type	Brain atrophy, thin corpus callosum	[7]
21	c.431A > G (p.Y144C)	F	20 days (died at 4 months)	Arab	Yes	Yes	Yes	Seizures of unknown type	Brain atrophy, thin corpus callosum	[7]
22	c.431A > G (p.Y144C)	F	25 days (died at 3.5 months)	Arab	Yes	Yes	Yes	Seizures of unknown type	Brain atrophy, thin corpus callosum	[7]
23	c.667C > T (p.R223C)	M	6 weeks (died at 10 months)	NA	Yes	Yes	Yes	multifocal clonic seizures and erratic myoclonic fits	NA	[8]
24	c.925G > A (p.G309S)		3 months (NA)	Korean	NA	Yes	NA	Seizures of unknown type	NA	[9]
25	c.989G > A (p.R330H), c.1113G > T (p.L371P)	F	6 weeks (alive at 16 years)	French	No	Yes	Yes	myoclonic focal and generalized seizures, refractory	Marked ventriculomegaly, abnormal T2 hyperintensities in the lentiform nuclei and dorsal brainstem	[10]
26	c.1256G > A (p.R419H), c.251A > C (p.H84P)	M	10 months (alive at 5 years)	French	No	Yes	Yes	Myoclonic generalized and focal seizures	Variably severe ventriculomegaly, Dentate nuclei, brainstem, and pallidal T2 hyperintensity	[10]
27	c.925G > A (p.G309S), c.943G > C (p.G315R)	M	1 day (died at 37 days)	Chinese	No	Yes	Yes	No epileptic seizures	cortical thinning, long T1 and long T2 signal shadows in the bilateral external capsule, enlargement of the ventricles	[11]
28	c.925G > A (p.G309S), c.943G > C (p.G315R)	F	1 day (died at 34 days)	Chinese	No	Yes	Yes	No epileptic seizures	Unclear corticomedullary demarcation of the bilateral cerebral hemispheres, enlargement of the ventricles, widened cerebral sulci and fissure	[11]
29	c.925G > A (p.G309S), c.550G > T (p.D184Y),	F	1 day (died at 2 months)	Chinese	NA	Yes	Yes	No epileptic seizures	NA	[22]
30	c.424G > T (p.D142Y), c.988C > T (p.R330C)	NA	NA	Chinese	NA	NA	NA	Generalized or secondary generalized tonic–clonic seizures, epileptic spasms, focal seizures, refractory	NA	[23]
31	c.479A > G (p.Q160R), c.727C > T (p.L243F)	NA	NA	Chinese	NA	NA	NA	Epileptic spasms, refractory	NA	[23]
**Hereditary spastic paraplegia**										
32	c.476 A > C (p.H159P), c.1255 C > T (p.R419C)	F	2 year (alive at 20 years)	North American	No	Yes	No	No epileptic seizures	Brain atrophy	[4]
33	c.476 A > C (p.H159P), c.1255 C > T (p.R419C)	F	1 year (alive at 17 years)	North American	No	Yes	No	No epileptic seizures	NA	[4]
34	c.1256G > A (p.R419H), 1269-76dup (p.S426*)	F	1 month (alive at 8 years)	French and Chinese	No	Yes	Yes	No epileptic seizures	mild ventriculomegaly	[10]
35	c.1255C > T (p.R419C), chr 6p21 del	F	2 months (alive at 5.5 years)	mixed European ancestry	NA	Yes	Yes	One seizure at 2 months of age after receiving vaccinations	Normal at 3 years	[12]
36	c.1255C > T (p.R419C), chr 6p25.1 del	M	6 weeks (alive at 14 years)	mixed European ancestry	NA	Yes	NA	several seizures before 6 weeks, no recurrence	Two small foci of T2/FLAIR signal in periventricular and deep white matter of right posterior frontal lobe at 13 years	[12]
37	c.424G > T (p.D142Y)	F	2 year (alive at 41 years)	Chinese	Yes	No	No	No epileptic seizures	Normal	[13]
38	c.424G > T (p.D142Y)	M	1 year (alive at 30 years)	Chinese	Yes	No	No	No epileptic seizures	Normal	[13]
39	c.424G > T (p.D142Y)	F	5 year (alive at 26 years)	Chinese	Yes	No	No	No epileptic seizures	Normal	[13]
40	c.424G > T (p.D142Y)	F	3 year (alive at 23 years)	Chinese	Yes	No	No	No epileptic seizures	Normal	[13]
41	c.461 C > T (p.A154V), c.1082 C > T (p.P361L)	M	6 months (alive at 19 years)	NA	No	Yes	Yes	Mild seizures only between 15 and 30 months of age	Signal abnormalities in anterior parts of mesencephalon Diffuse brain atrophy	[14]
42	c.521_523del (p.V174del), c.1082 C > T (p.P361L)	F	10 months (alive at 15 years)	NA	NA	Yes	Yes	No epileptic seizures	Signal abnormalities in tegmentum and periaqueductal grey matter Mild progressive cerebellar atrophy	[14]
43	c.646 C > T (p.Q216*) c.407 C > A (p.P136H)	M	2.5 year (alive at 9 years)	Northern European and Ashkenazi Jewish	No	No	NA	No epileptic seizures	Abnormal signal hyperintensities in the bilateral dentate nuclei	[15]
44	c.422G > A (p.G141E) chr6: del	M	18 months (alive at 13 years)	Irish	No	NA	NA	No epileptic seizures	Normal	[16]
45	c.422G > A (p.G141E) chr6: del	F	24 months (alive at 7 years)	Irish	No	NA	NA	No epileptic seizures	Normal	[16]
46	p. D364G	F	NA (alive at 9 years)	NA	NA	NA	NA	NA	NA	[17]
47	c.1082 C > T (p.P361L) ex. 1–2 del	M	5 year (alive at 22 years)	Czech Roma	No	No	NA	No epileptic seizures	Normal	[18]
**Juvenile-onset epilepsy**										
48	c.253C > G (p.P85A) c.403 C > G (p.H135D)	F	8 years (died at 15 years)	NA	No	Yes	No	GTC, SE, focal seizures, progressive myoclonus	Extensive signal abnormalities in left caudate, cortex and cerebellum	[19]
49	c.589G > A (p.V197M) c.1205 T > C (p.F402S)	M	12 years (died at 20 years)	Chinese	No	No	Yes	generalized, partial and/or secondary generalized convulsive status epilepticus	Increased wandering lesions involving bilateral frontal, temporal, and parietal lobes, occipital cortex and subcortical	[20]
50	c.589G > A (p.V197M) microdeletion	F	16 years (alive at 17 years)	NA	No	No	No	super refractory focal motor status epilepticus	Restricted diffusion in the cortical-subcortical areas of the right frontal lobe, right insula, right thalamus and to lesser extent in the right temporal, both parietal lobes and left frontal lobe	[21]
**Onset or phenotype unknown**										
51	c.1275G.C (p.L425L), c.1277C.T (p.S426F)	NA	NA	NA	NA	NA	NA	NA	NA	[3]
52	c.431A > G (p.Y144C) c.530 T > A (p.V177D)	F	died at 2 days	Arab	Yes	NA	Yes	No	NA	[7]
53	c.925G > A (p.G309S) c.457A > G (p.R153G)	F	NA	Hispanic	NA	NA	Yes	NA	NA	[7]
54	c.467 C > T (p.T156M) c.905-1G > A (splice-acceptor)	F	NA	NA	NA	NA	NA	NA	Epileptic encephalopathy	[25]
55	c.467 C > T (p.T156M) intragenic deletion	NA	NA	NA	NA	NA	Yes	NA	Signal abnormalities in dentate nuclei	[26]
56	c.989G > A (p.R330H), c.1113G > T (p.L371P)	NA	NA	NA	NA	NA	Yes	NA	Signal abnormalities in putamen, dentate nuclei	[26]
57	c.1256G > A (p.R419H), c.251A > C (p.H84P)	NA	NA	NA	NA	NA	Yes	NA	Signal abnormalities in caudate nuclei, putamen, dentate nuclei	[26]
58	c.1256G > A (p.R419H), 1268-69ins (p.T424*)	NA	NA	NA	NA	NA	Yes	NA	NA	[26]

*M* male, *F* female, *MRI* magnetic resonance imaging, *NA* data not available, *GTC* generalized tonic–clonic seizures, *SE* status epilepticus

**Fig. 6:**
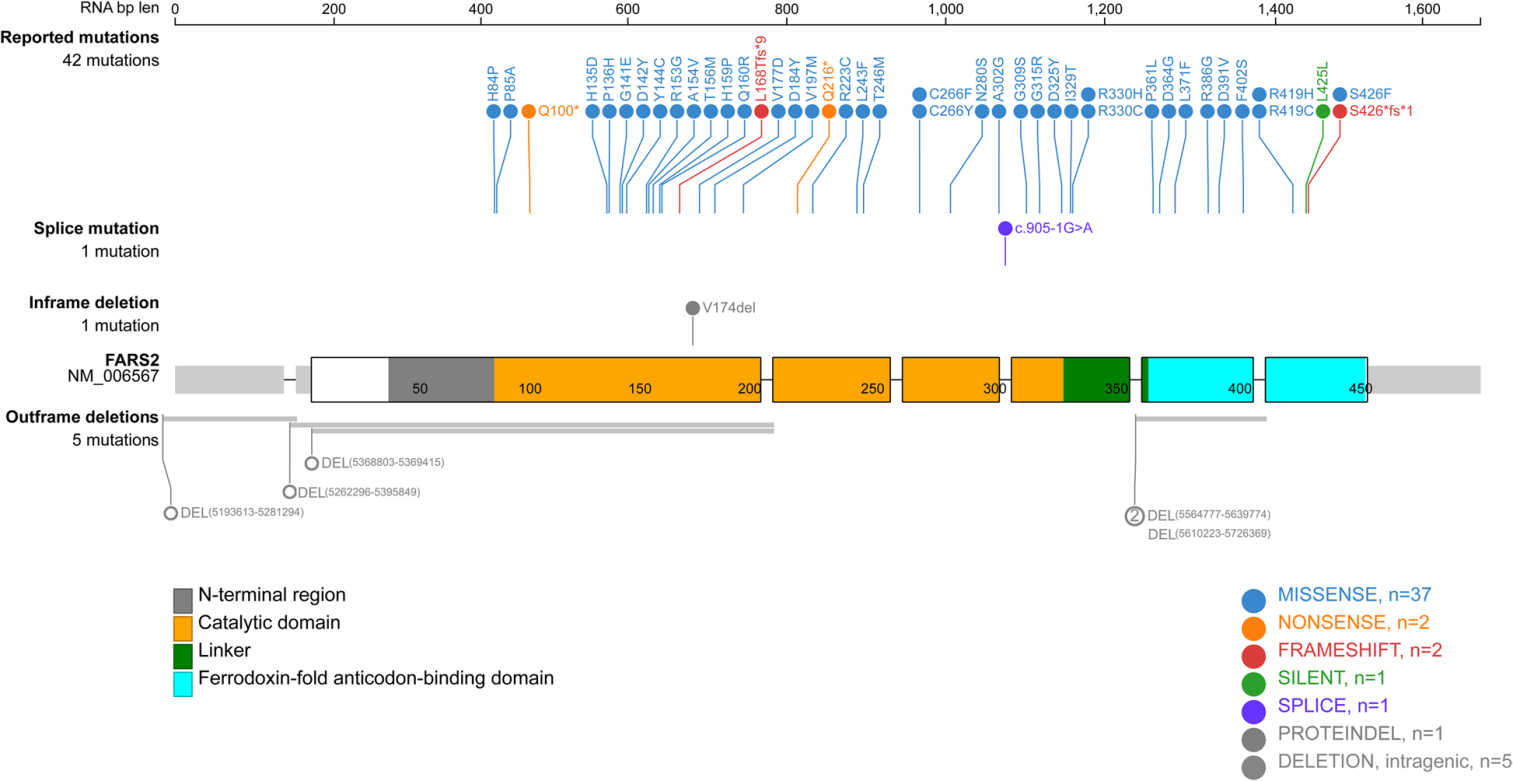
The mutations include 37 missense mutations, 2 nonsense mutations, 2 frameshift deletions, 1 silent mutation, 1 splice-site mutation, and 1 in-frame and 5 out-of-frame deletions

We summarized all the variations in FARS2 that have been reported to date (Table 2). These mutations included 37 missense mutations, 2 nonsense mutations, 2 frameshift deletions, 1 silent mutation, 1 splice-site mutation, and 1 in-frame and 5 out-of-frame deletions. We generated lollipop plots to visualize FARS2 mutations using Protein-Paint (Fig. 6). Patients carry these mutations in an autosomal recessive manner: compound heterozygous with two mutations or homozygous with one mutation.

 The most commonly reported variant is Y144C, which was observed in ten families with homozygous status and an additional family with heterozygous status, and all reported families with this variant are Arab. The second most common variant is G309S, with three homozygous Korean families, two heterozygous Chinese families, and one heterozygous Hispanic family. The variant D142Y was found in only one homozygous and one heterozygous Chinese family. A literature review revealed regional differences in the gene variants of COXPD14.

Most of the early-onset epileptic phenotypes (24/31) occurred in Asians, especially Arabs (14/24), and neither of the other two phenotypes were found in Arabs (Table 3). COXPD14 is inherited in an autosomal recessive manner; more than half of the early-onset epileptic phenotypes (15/26) were born from consanguineous parents. In contrast, in the hereditary spastic paraplegia phenotype, these conditions are rare in the Asian population, and most were nonconsanguineous.

**Table 3:** Clinical feature of reported FARS2 subjects [2–23]

	early-onset epileptic encephalopathy	hereditary spastic paraplegia	juvenile onset epilepsy
Number	31	16	3
Male: female	11:18	6:10	1:2
Ethnicity, Asian	24/31	4/13	1/3
Consanguinity	15/26	4/12	0
Onset age	1 day-10 month	1 month-5 years	8-16 years
Alive	10/27	16/16	1/3
Developmental delay	29/29	7/13	1/3 (motor and speech)
seizures	28/31	3/15	3/3
Heterozygous	11/31	11/16	3/3
Brain atrophy	18/23	3/14	2/3
Thin corpus callosum	12/23	0	0
Hepatic disease	14/23	0	0
Increased transaminases	15/22	4/6	0
Elevated lactate	23/24	4/10	1/3
Increased CSF lactate	9/9	2/4	0

The age of onset in subjects with the early onset epileptic phenotype (*n* = 31) was from birth to six months, in comparison the age of onset from one month to five years in subjects with the hereditary spastic paraplegia phenotype (*n* = 16) and the age of onset from eight to 15 years in those with the juvenile-onset epilepsy phenotype (*n* = 3).

All individuals with the hereditary spastic paraplegia phenotype were alive at the time of reporting and showed long-term survival with an age range between 5.5–41 years, compared to more than 60% of subjects (17/27) with the early-onset epileptic phenotype having died at the time of reporting.

The epileptic phenotype of FARS2 deficiency was the most severe. Three patients died within the first two months of life, and no seizures were observed; all the remaining patients (28/28) suffered from epilepsy, and most of them started in the first 6 months of life. Developmental delay was observed in all subjects (29/29) mentioned in the literature with early-onset epileptic phenotypes, while less severe developmental delay was observed in subjects with the other phenotypes.

Brain MRI showed a wide range of abnormalities including diffuse brain atrophy (18/23), thin corpus callosum (12/23), lesions in the dentate nuclei and cerebellum, and signal abnormalities in the putamen, caudate nucleus, and white matter. Diffuse cortical and subcortical atrophy is a common finding, especially in later stages of the disease. However, this is considered a nonspecific finding because it is similar to most advanced neurometabolic diseases. Thinning of the corpus callosum was observed in the early-onset epileptic phenotype but not in other phenotypes. A thin corpus callosum was found in our adult-onset patient, indicating that it may appear in late-onset epilepsy phenotypes of COXPD14, which reflects the markedly reduced cerebral white matter volume.

Of the previously reported cases with the early onset epilepsy phenotype, more than half of the subjects (14/23) had evidence of liver disease, and there was a consistent elevation of lactic acid levels in almost all subjects (23/24). CSF lactate levels were available for nine subjects and were elevated in all patients. In contrast, none of the subjects with the other two phenotypes showed evidence of liver disease.

## Discussion

Here, we report one FARS2 deficiency patient manifesting as adult-onset status epilepticus. We found an autosomal recessive mutation and identified a compound heterozygous FARS2 variant, c.467C > T (p.T156M), which is known and has been reported previously, while c.119_120del (p.E40Vfs*87) is a novel variant. The clinical phenotype of the patient was different from that reported in the literature. Unfortunately, the patient was not tested for protein function owing to financial constraints. Nevertheless, the p.T156M variant represented as COXPD 14 has been reported; according to the HGMD pro database [24] and ACMG guidelines [27], we considered the complex heterozygous variation in this patient to be theoretically pathogenic.

The type of COXPD14 was confirmed based on the main clinical findings combined with the age at onset because patients can develop identical symptoms at different ages, such as seizures, cognitive delay, decline in activities of daily living, and increased lactate levels [11]. Our report demonstrates that the age spectrum of epileptic phenotype onset extends to adulthood, except for early or juvenile onset. We are inclined to define the phenotypes based on the clinical manifestation rather than the age of onset, as in Elise Vantroys’ proposal, i.e., (ɪ) epileptic phenotype and (ɪɪ) spastic paraplegia phenotype [14].

In the previously reported cases, we found that three patients diagnosed with early onset epileptic encephalopathy were not accompanied by epilepsy, and the phenotype can still be called early-onset epileptic encephalopathy. The 3 patients without epilepsy died during the first two months of life, and no seizures were observed. Early-onset epileptic encephalopathy is characterized by seizures with an onset in the first 6 months of life [5–7], so we classified these 3 patients as having an epileptic phenotype. Interestingly, all three patients were Chinese and carried c.925G > A (p.G309S), which was found only in Asian populations. Patients with heterozygous mutations carrying this variant have a poor prognosis, whereas those with homozygous mutations have a relatively good prognosis [6].

Barcia et al. [10] reported a case of early-onset encephalopathy without epilepsy presenting with axial hypotonia and developmental delay within 1 month after birth. At age 8, the patient was unable to walk unassisted and had spastic paralysis, dystonic movements, and axial hypotonia. In addition, the EEG was normal, and MRI showed no cortical atrophy or encephalopathy-like changes; therefore, we consider that this patient should be classified as having a spastic paraplegia phenotype rather than an early-onset encephalopathy phenotype, as suggested by Giulia Barcia et al. (Patient 34 in Table 2).

The earliest case of COXPD14 was an Arab girl carrying a homozygous c.431A > G (p.Y144C) mutation. Since then, 13 cases of this homozygous mutation have been reported successively in Arab populations, whereas it has not been reported in any other Asian populations, indicating significant regional differences in COXPD14 cases.

The epileptic phenotype of COXPD14 is the most severe, consisting of epileptic encephalopathy and diffuse cortical dysfunction. Most patients with early-onset epileptic encephalopathy die before the age of age [2, 3, 6]. To date, only three cases of juvenile-onset epilepsy have been reported [19–21], and the patient with the longest survival time was a Chinese patient with novel compound heterozygous mutations (p.V197M and p.F402S) who died within 3 years after onset due to respiratory failure caused by pulmonary infection at the age of 20. Our patient had normal growth and development before the age of 27 years. What is even more interesting is that each seizure she experienced was status epilepticus. Neither the age of onset nor the epileptic phenotype have been previously reported.

Most cases of status epilepticus in adults are due to an underlying structural brain lesion or toxic or metabolic disturbance [28]. If the underlying medical or structural cause is of recent origin (< 1 to 2 weeks), status epilepticus is referred to as acute symptomatic or “provoked”. In adults, the most common etiology is acute symptoms, accounting for approximately half of all cases. In our patient, the onset of the disease was acute symptomatic with status epilepticus, without other epilepsy manifestations, and each time presented with refractory status epilepticus, with clear triggers such as fatigue, mood swings, and lack of sleep, and the treatment required sedative drugs and respiratory support. In COXPD14, seizures of the early-onset epileptic encephalopathy phenotype are difficult to control and may progress quickly to intractable seizures with frequent status epilepticus at an early age [29]. The patient we report here presented solely with status epilepticus, which is quite rare in COXPD14. Metabolic epilepsies, including status epilepticus, are the main neurological manifestations of mitochondrial diseases such as MELAS, MERFF, or POLG-related disorders [30]. For our patient, considering the dynamic changes in MRI (Fig. 2), the cortical energy metabolism disorder should be the underlying cause of status epilepticus with additional specific causes.

Studies have found that mt-aaRS gene mutations are mostly related to central nervous system diseases that can be classified into four categories: leukoencephalopathy, early brain disease, infantile fatal neurodegenerative syndrome, and sensory nerve abnormalities [31]. The basal ganglia nuclei and white matter of the central nervous system are more vulnerable, as in COXPD14. However, previously reported cases have also shown cortical atrophy, especially in early-onset epileptic encephalopathy, which is usually accompanied by seizures and regression [5, 6, 10, 11]. In addition to cortical atrophy, MRI of our patient also showed a thin corpus callosum, which was previously reported as an early-onset epileptic phenotype [4–7]. The thin corpus callosum mostly appeared in the Asian population (10/12), and no clear correlation of mutation sites was found, which may be related to ethnic differences.

Our patient had no basal ganglia abnormalities but presented with cortical gyri-like abnormal signals similar to mitochondrial encephalopathy, and the abnormal signals disappeared on repeated MRI one month later, consistent with the characteristics of mitochondrial metabolism. However, cerebral perfusion imaging revealed that the original lesion was hypoperfusion, which is different from the hyperperfusion of mitochondrial encephalopathy and may be caused by different biochemical mechanisms.

More interestingly, the functional imaging examination of our patient showed CCD, which refers to a decrease in blood flow, glucose oxidation metabolism level, and even crossed cerebellar atrophy in the opposite cerebellar hemisphere when one side of the cerebral hemisphere is diseased. CCD can appear in patients with status epilepticus, which is mainly related to chronic focal epilepsy and may be related to additional cross-nerve excitatory toxicity damage [32–34]; it has not been reported in patients with FARS2 gene mutations. The initial PET-CT and MRI of the index patient showed abnormal signals in the opposite cerebellum of the epileptogenic focus, considered CCD (Figs. 2 and 3). Repeated MRI and PWI showed that the abnormal signals in the cerebellum disappeared and the perfusion was normal, whereas the perfusion of the probable epileptogenic focus was not reinstated (Fig. 4), indicating that the CCD might be a secondary change that could be recovered in a short time after the initial cause was removed. The same dynamic changes in CCD in COXPD14 have not been previously reported. Unfortunately, positron emission tomography-CT was not performed to confirm these changes.

On the basis of the clinical symptoms and imaging characteristics of this patient, we initially suspected a special type of mitochondrial encephalopathy, and no abnormal mutation sites were found after the completion of mitochondrial gene testing. Finally, WES identified a mutation in the FARS2 gene and confirmed the diagnosis. Our study highlights that genetic testing, including WES, plays an important role in the diagnosis of diseases with multiple phenotypes, especially in the differential diagnosis of diseases, and can be used as the gold standard in the diagnosis of diseases. WES is better than immunohistochemical assays of muscle biopsy and measurement of lactate, amino acids, and organic acids in the blood and urine. We believe that our report verifies and may expand the epileptic phenotype and genotypic spectrum of COXPD14, providing clinical evidence that may contribute to subsequent studies or experiments on COXPD14 in adults.

## Data Availability

The datasets generated and/or analysed during the current study are available in the Sequence Read Archive (SRA) repository, [SRR26937199].
